# Out of the ice age: Preservation of cardiac allografts with a reusable 10 °C cooler

**DOI:** 10.1016/j.xjon.2024.08.005

**Published:** 2024-08-21

**Authors:** John M. Trahanas, Timothy Harris, Mark Petrovic, Anthony Dreher, Chetan Pasrija, Stephen A. DeVries, Swaroop Bommareddi, Brian Lima, Chen Chia Wang, Michael Cortelli, Avery Fortier, Kaitlyn Tracy, Elizabeth Simonds, Clifton D. Keck, Shelley R. Scholl, Hasan Siddiqi, Kelly Schlendorf, Matthew Bacchetta, Ashish S. Shah

**Affiliations:** a Department of Cardiac Surgery, Vanderbilt University Medical Center, Nashville, Tenn; b Department of Cardiology, Vanderbilt University Medical Center, Nashville, Tenn; c Department of Biomedical Engineering, Vanderbilt University, Nashville, Tenn

**Keywords:** heart transplant, static cold storage, primary graft dysfunction, allograft preservation

## Abstract

**Objective:**

Static cold storage with ice has been the mainstay of cardiac donor preservation. Early preclinical data suggest that allograft preservation at 10 °C may be beneficial. We tested this hypothesis by using a static 10 °C storage device to preserve and transport cardiac allografts.

**Methods:**

In total, 52 allografts were recovered between July 2023 and March 2024 and transported using a 10 °C storage cooler. Results were compared to a 3:1 propensity match of allografts transported on ice. Patients were excluded for the following reasons: dual viscera transplant, previous heart transplant, complex congenital heart disease, or allograft injury during procurement.

**Results:**

Among the 10 °C cooler cohort, median total ischemic time was 222 minutes at 10 °C versus 193 minutes on ice (*P* < .0001). Intraoperative change in lactate was statistically lower at 10 °C (3.6 vs 5.1 mmol/L, *P* = .0016). Cardiac index score was greater in 10 °C cooler hearts at 24 (3.2 vs 3.0, *P* = .016) and 72 hours (3.3 vs 2.9, *P* = .037), despite similar vasoactive inotrope scores. There was no difference in severe primary graft dysfunction (1.9 vs 2.6%, *P* > .99). 10 °C hearts demonstrated less change in lactate but no difference in vasoactive inotrope scores or cardiac index. In hearts with extended ischemic time, delta lactate was lower in 10 °C cooler hearts. There was no statistical difference in outcomes for donor hearts >40 years old.

**Conclusions:**

This is an early experience of static preservation in a 10 °C cooler. Postoperative allograft function was excellent, and lactate profiles lower in those allografts with extended ischemic times. Static cold storage targeting 10 °C may offer an inexpensive method for extended heart preservation. Further investigation is needed to assess long-term outcomes of 10 °C storage.

## Graphical Abstract

**Figure:**
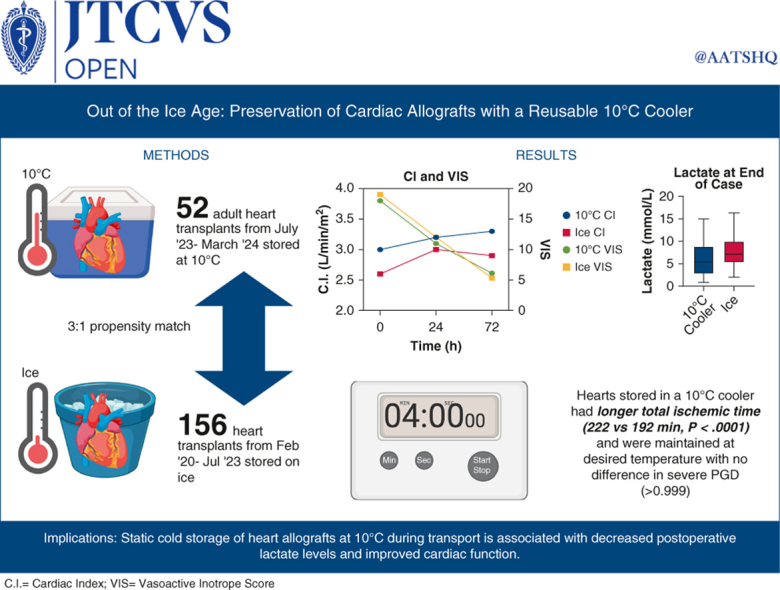

**Figure:**
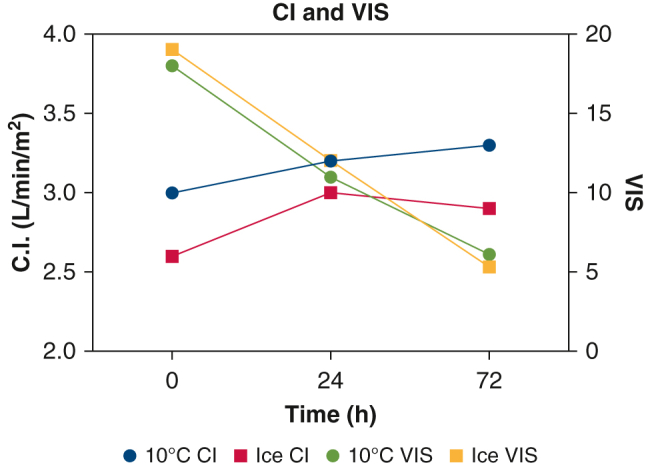
*CI*, Cardiac index; *VIS*, vasoactive inotrope score.

**Central Message**

Hearts stored in a 10 °C cooler demonstrate improved cardiac index postoperatively compared with hearts stored on ice despite similar inotrope requirements, as well as have increased lactate clearance.

**Perspective**

There is renewed interest in heart allograft preservation temperature to reduce the incidence of ischemia reperfusion injury. Our data demonstrate that adult DBD and DCD heart allografts stored statically in a 10 °C cooler demonstrate increased cardiac function 24 and 72 hours after transplant, as well as lower lactate levels despite prolonged preservation time.

Severe primary graft dysfunction (PGD) after heart transplantation impacts approximately 10% of patients after heart transplantation.^1^ The incidence of PGD has remained relatively stagnant over the past decade and remains a primary source of morbidity and mortality after heart transplantation, with an associated incidence of 7% 1-year mortality for all patients and a 38% incidence of 1-year mortality among those with severe PGD.^2^

Myocardial ischemia during donor storage is a known, important cause of PGD after heart transplantation.^3^ Although there have been extensive studies on the recovery process (eg, duration of storage, type of perfusate, static vs dynamic storage), there is a limited understanding of the ideal temperature for static storage. Most used in clinical practice is storage on ice, which reduces metabolic demand to 10% of baseline activity.^4^ However, prolonged storage on ice has been associated with cellular edema, mitochondrial dysfunction, and subsequent graft failure.^5^, ^6^, ^7^, ^8^, ^9^ In addition, a second injury during reperfusion has been demonstrated in hearts stored on ice.^10^

Within the realm of lung transplantation, recent studies have shown that 10 °C static cold storage (SCS) allows for dramatically longer cold ischemic time with less pulmonary dysfunction compared with storage on ice.^11^ In addition, preclinical studies demonstrate that 10 °C SCS facilitates improved function in injured allografts.^12^ These studies have resulted in a disruptive change of clinical practice in lung recovery and transport at many high-volume centers and have triggered a clinical trial evaluating prolonged 10 °C storage.

Heart allografts may obtain similar benefit and longer preservation times from warmer temperatures. The Global Utilization and Registry Database for Improved Heart Preservation registry data demonstrates a reduction in severe PGD and 1-year survival when cardiac allografts are stored at 4 to 8 °C.^13^ Taking into account the experience in lungs, preclinical data, and the beneficial effect of warmer temperatures in the Global Utilization and Registry Database for Improved Heart Preservation registry, we have changed our practice to target storage of hearts at 10 °C using the TRAFEROX system, an insulated cooler designed for lung preservation that is set to maintain an internal temperature of 10 °C (Figure 1). This provides an additional opportunity to understand how prolonged non-ice SCS affects heart allograft function.

**Figure 1:**
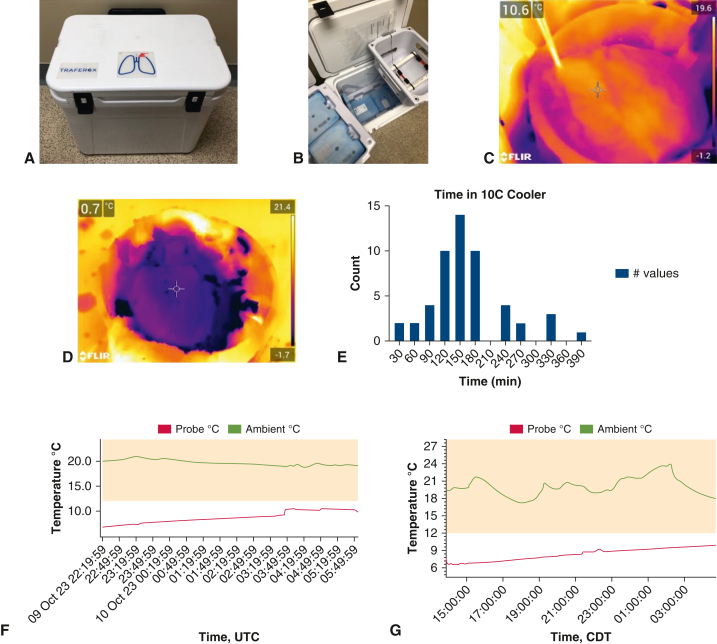
Cooling and maintenance at 10 °C. A and B, 10 °C storage cooler and packs. C, Thermography of heart after storage in the cooler. D, Thermography of heart after storage on ice. Coloration demonstrates a relative temperature to objects within the frame, whereas *purple* is cooler, *red* is moderate, and *yellow* is warmer temperatures relative to objects in the frame. The scale on the *right* side of the image displays the range of temperatures within this unique frame. E, Distribution of time hearts were stored in 10 °C storage cooler. F and G, Temperature curve of a heart using the cooler for <4 and >4 hours, respectively, both demonstrating cooling and maintenance of temperature at 10 °C. *UTC*, Universal Time Zone; *CDT*, Central Daylight Time.

We reviewed our experience using this 10 °C storage device. We examined preoperative, intraoperative, and postoperative metrics, with a particular interest in early postoperative hemodynamics and the rate of severe PGD, as it compared with our previous use of SCS with ice. Furthermore, we investigated whether extended preservation time greater than 4 hours, and preservation of older donors, at 10 °C would demonstrate improved outcomes compared to ice SCS.

## Methods

### Data Collection

**Figure 2:**
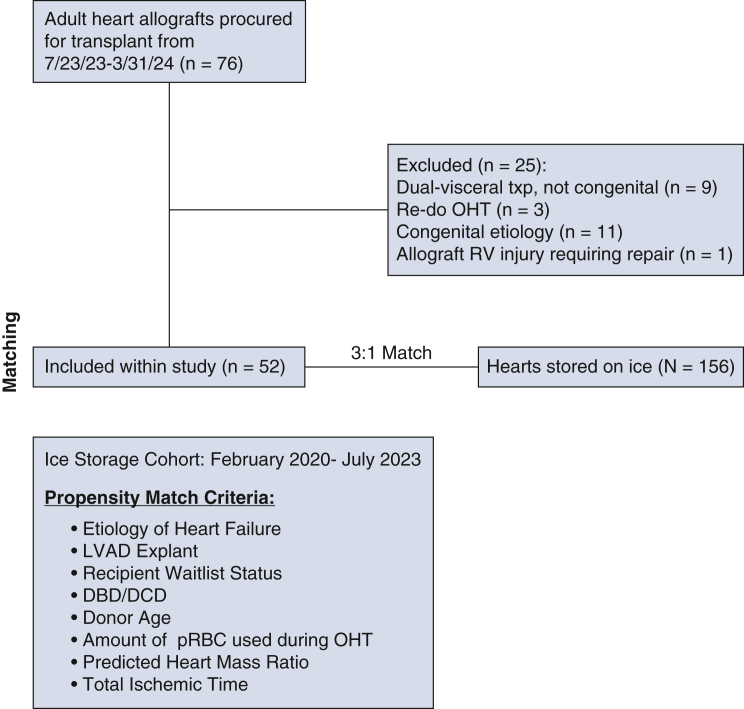
Inclusion criteria for 10 °C cooler evaluation. *OHT*, Orthotopic heart transplant; *RV*, right ventricle; *LVAD*, left ventricular assist device; *DBD/DCD*, donation after brain death/donation after circulatory death; *pRBC*, packed red blood cells.

**Figure 3:**
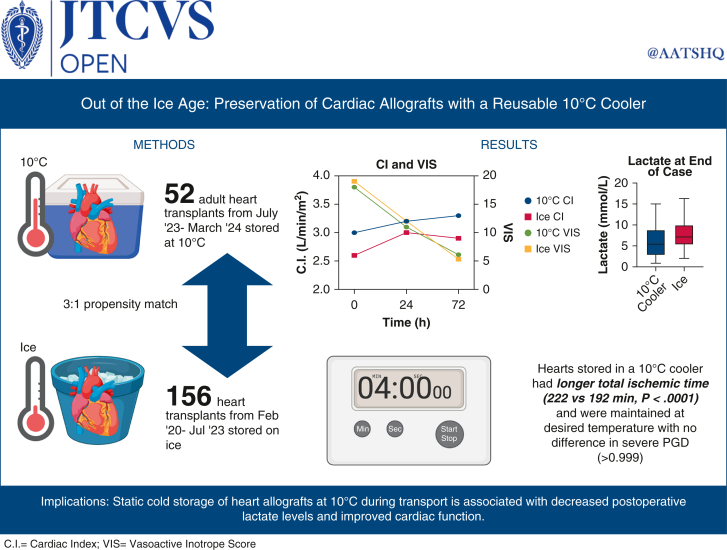
Description of CI, VIS, and lactate of hearts stored at 10 °C compared to historical controls stored on ice.

We retrospectively reviewed all adult heart transplants between July 2023 and March 2024 at a single university transplant center. All adult patients who received a heart allograft stored using the 10 °C cooler were included. The following exclusion criteria were applied: retransplant, dual-organ transplant (ie, heart-liver, heart-kidney, heart-lung), or complex congenital etiology of heart failure (Figure 2). An additional patient was excluded as the result of an injury to the allograft during procurement, which required ligation of the acute marginal artery and patch repair. A 3:1 propensity match was performed with all adult heart transplant recipients who received allografts stored on ice between February 2020 and June 2023, using the same exclusion criteria (Figure 3). We used February 2020 as the starting time point for the historical cohort, which is when our institution began procuring donation after circulatory death (DCD) hearts. The propensity match included the following cohort parameters: etiology of heart failure (ischemic vs nonischemic), left ventricular assist device explant, recipient waitlist status, total ischemic time, predicted heart mass (PHM) ratio, donor age, number of packed red blood cells used during transplant, and type of recovery (donation after brain death [DBD] or DCD). They were optimized for balance, included in the calculation of the generalized Mahalanobis distance between patient groups, and considered in the estimation of the propensity score. A genetic algorithm was then applied to select the optimal scaling factors for each covariate, with the goal of maximizing the smallest *P* value in the covariate balance tests. Donor heart characteristics were obtained through the United Network for Organ Sharing database. Recipient heart characteristics were procured from electronic medical records. The institutional review board at Vanderbilt University Medical Center approved the study protocol and publication of data (211058, expires June 20, 2025). Written consent for publication of study data was waived by the institutional review board because of the retrospective nature of study. This study complies with The American Association for Thoracic Surgery's Publication Ethics statement.

Temperature data of heart allografts stored in the 10 °C cooler were obtained from the 10 °C storage cooling system. Additional thermographic data were obtained with a thermal imaging camera (T530; FLIR) at 2 time intervals: the time of explant at the donor site and the time the allograft was removed from storage at the recipient hospital before implantation. Thermal imaging was used exclusively for the purpose of the study to assess myocardial tissue and correlate tissue temperature with cooler data in order to assess device accuracy.

### End Points

**Figure E1:**
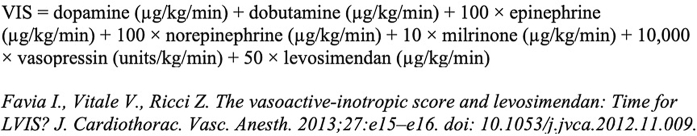
Vasoactive inotrope score (*VIS*) equation.

Our primary outcome of interest was evidence of severe PGD, as defined by the International Society of Heart and Lung Transplantation 2014 Consensus document.^14^ Patients who required venoarterial extracorporeal membrane oxygenation support within the first 24 hours postoperatively were identified in the medical record. We reviewed additional outcomes including serum lactate changes intraoperatively, the calculated vasoactive inotrope score (VIS, which includes the following pressors: dobutamine, dopamine, milrinone, epinephrine, norepinephrine and vasopressin; see Figure E1 for list of pressors and equation), dependency on pacing, and cardiac index (CI) at the following 3 time points: immediately postoperatively upon arrival to the intensive care unit (ICU) and at 24 and at 72 hours from the end of the case. ICU and hospital length of stay (LOS) were additionally measured.

### Data Analysis

Continuous variables are described as medians with interquartile ranges; categorical variables are reported as numbers and percentages. Given sample size, nonparametric tests were used. Continuous variables were compared using a Mann-Whitney *U* test; categorical variables were compared using a Fisher exact test. All statistics were performed in GraphPad Prism, version 10.2.2.

## Results

### Donor and Recipient Characteristics

From July 2023 to March 2024, 76 adult cardiac allografts were stored in the 10 °C cooler and transplanted at Vanderbilt University Medical Center. In total, 52 heart transplants were included in this study after applying the aforementioned exclusion criteria. A 3:1 propensity match was performed providing a control cohort of 156 hearts that were stored on ice.

Donor characteristics, stratified by storage temperature, are outlined in Table 1. Compared with hearts stored on ice, there was no difference in median donor heart age (34 years vs 31, *P* = .09), sex (34.6% female vs 29.5% female, *P* = .49), or PHM ratio (1.0 vs 0.94, *P* = .07). There was no difference in comorbidities such as hypertension, diabetes, as well as ABO group; however, there was a statistically greater number of donors with a hepatitis C virus (HCV) nucleic acid test positive (+) result in the ice group (1.9% vs 21.2%, *P* = .0004). Donor heart cardiac arrest with cardiopulmonary resuscitation (44.2% vs 62.8%, *P* = .02) and anoxia/hypoxia as the cause of death (36.5% vs 53.8% *P* = .037) was significantly more prevalent in the ice group, and the preprocurement left ventricular ejection fracture was significantly lower in this group (65% vs 60% *P* = .02). There was a statistically significant difference with a greater percentage of DBD procurements for hearts stored on ice (50% vs 69.9%, *P* = .01).

**Table 1:** Donor characteristics

Variable	10 °C cooler (n = 52)	Ice (n = 156)	*P* value
Sex			
Female	18 (34.6%)	46 (29.5%)	.49
Male	34 (65.4%)	110 (70.5%)	
Median age, y	34 [26-40]; (17-52)	31 [25-39]; (12-54)	.09
>40	12 (23.1%)	31 (19.9%)	.69
Median BMI	29 [24-34] (16-69)	27 [23-33]; (17-58)	.24
Blood group			
A	11 (21.2%)	54 (34.6%)	.08
B	5 (9.6%)	10 (6.4%)	.54
AB	1 (1.9%)	2 (1.3%)	.99
O	35 (67.3%)	90 (57.7%)	.25
HbA1c	5.4 [5.1-5.7]; (4.5-8.8)	5.4 [5.1-5.6]; (3.8- 9.2)	.90
Not available	11 (21.2%)	32 (20.5%)	
History of hypertension			
No	37 (71.2%)	118 (75.6%)	.23
Yes	13 (25%)	26 (16.7%)	
Unavailable	2 (3.8%)	12 (7.7%)	
HCV NAT+‡	1 (1.9%)	33 (21.2%)	.0004∗
Coronary angiogram			
Coronary angiogram performed	18 (34.6%)	59 (37.1%)	.74
No coronary stenosis	14 (77.8%)	45 (76.3%)	.99
Nonsignificant coronary stenosis	4 (22.2%)	14 (23.7%)	
Cardiac arrest and CPR, n (%)	23 (44.2%)	98 (62.8%)	.02†
Cause of death			
Intracranial hemorrhage	7 (13.5%)	20 (12.8%)	.63
Trauma	12 (23.1%)	26 (16.7%)	.41
Anoxia/hypoxia	19 (36.5%)	84 (53.8%)	.037†
Cerebrovascular/ischemic stroke	1 (1.9%)	0	.25
Other	13 (25%)	26 (16.7%)	.22
Median LVEF, n (%)	65 [60-68]; (40-76)	60 [59-65]; (40-75)	.02†
Donation type			
DBD	26 (50%)	109 (69.9%)	.01†
DCD + TA-NRP	26 (50%)	47 (30.1%)	

*BMI*, Body mass index; *HbA1c*, hemoglobin A1C; *HCV NAT+*, hepatitis C virus nucleic acid test positive result; *CPR*, cardiopulmonary resuscitation; *LVEF*, left ventricular ejection fraction; *DBD*, donation after brain death; *DCD + TA-NRP*, donation after circulatory death + thoraco-abdominal normothermic regional perfusion.

∗ *P* < .001.

† *P* < .05.

‡ *P* < .01.

There were no differences in recipient sex, blood type, or body mass index (Table 2). Recipients who received allografts stored on ice were statistically older (55 vs 59 years, *P* = .02). There was no difference in PHM ratio, waitlist status, or female heart-male recipient ratio. Etiology of heart failure as an indication for transplant (26.9% vs 35.9% *P* = .31), previous sternotomy (36.5% vs 32.1% *P* = .42), and left ventricular assist device explant (48.1% vs 56.4% *P* = .61) were not statistically different between groups.

**Table 2:** Recipient characteristics

Variable	10 °C cooler (n = 52)	Ice (n = 156)	*P* value
Sex			
Female	17 (32.7%)	39 (25%)	.28
Male	35 (67.3%)	117 (75%)	
Median age, y	55 [46-62]; (19-69)	59 [51-66]; (20-73)	.02∗
Median BMI	30 [25-35]; (18-41)	30 [24-33]; (17-44)	.59
Blood group			
A	14 (26.9%)	60 (38.5%)	.18
B	7 (13.5%)	17 (10.9%)	.62
AB	1 (1.9%)	6 (3.8%)	.68
O	30 (57.7%)	73 (46.8%)	.20
Median PHM ratio	1.0 [0.89-1.2]; (0.69-1.6)	0.94 [0.84-1.1]; (0.65-1.7)	.07
Indication for transplant			
Ischemic cardiomyopathy	14 (26.9%)	56 (35.9%)	.31
Nonischemic cardiomyopathy	38 (73.1%)	100 (64.1%)	
Waitlist status			
1	6 (11.5%)	11 (7.1%)	.38
2	14 (26.9%)	46 (29.5)	.86
3	11 (21.2%)	32 (20.5%)	.99
4	12 (23.1%)	39 (25%)	.85
5	0	0	
6	9 (17.3%)	28 (17.9%)	.99
Previous sternotomy	25 (48.1%)	88 (56.4%)	.42
LVAD explant	19 (36.5%)	50 (32.1%)	.61
Female donor-male recipient	5 (9.6%)	24 (15.4%)	.36

*BMI*, Body mass index; *PHM*, predicted heart mass; *LVAD*, left ventricular assist device.

∗ *P* < .05.

### Hearts Are Able to Be Maintained at 10 °C During Transport Even During Prolonged Ischemic Time

During procurement, hearts were flushed with cold del Nido cardioplegia, procured in standard fashion, and stored in cold del Nido solution. After packaging in 3-layer bag and plastic container, hearts were placed in a 10 °C storage cooler (Figure 1). Hearts achieved a median temperature of 9.6 °C (±1.0 °C) at the end of storage. A representative temperature curve of a stored heart is depicted in Figure 1. Hearts were stored for a median of 153 minutes in the cooler, the longest for 382 minutes. For hearts stored >4 hours within the cooler, average final temperature of hearts was 9.85 °C, demonstrating that the 10 °C storage cooler was able to maintain the desired temperature even for extended preservation time (Table E1).

Thermographic images were captured in a subset of these hearts (T530; FLIR). A representative image of a heart at the end of storage at 10 °C is demonstrated in Figure 1. Comparatively, a thermographic image of a heart stored on ice is shown. Thermographic images were consistent with temperatures from the 10 °C storage cooler (Table E2).

### Hearts Stored at 10 °C Demonstrate Improved Intraoperative and Postoperative Outcomes Despite Longer Storage Time

There was a statistically significant increase in median total ischemic time between hearts stored at 10 °C (222 minutes vs 192 minutes, *P* < .0001) (Table 3). In addition, 44.2% of hearts stored at 10 °C had an ischemic time greater than 4 hours, compared with 12.8% of allografts stored on ice (*P* < .0001). Intraoperatively, there was a statistically lower difference in the change in lactate (delta lactate) in the 10 °C cooler cohort, defined as the difference in levels between the removal of the aortic clamp and the end of the case (3.6 vs 5.1 mmol/L, *P* = .0016).

**Table 3:** Preservation and intraoperative findings

Variable	10 °C cooler (n = 52)	Ice (n = 156)	*P* value
Donor distance, NM	332 [160-507]; (0-1683)	249 [139-442]; (0-966)	.12
Time in cooler, min	153 [121-184]; (20-382)	n/a	
Preservation temperature, °C	8.8 [8.3-9.4]; (6.2-10)	n/a	
Mean graft temperature at time of removal, °C	9.6 [9.0-10]; (6.1-11)	n/a	
Total donor heart ischemic time, min	222 [200-288]; (44-511)	192 [168-221]; (49-377)	<.0001∗
0-240	29 (55.8%)	136 (87.2%)	
>240	23 (44.2%)	20 (12.8%)	<.0001∗
Duration of cardiopulmonary bypass, min	130 [108-159]; (77-253)	133 [108-161]; (74-338)	.99
Number of pRBCs transfused			.29
0-4 units	42 (80.1%)	109 (70.0%)	
5-9 units	6 (11.5%)	31 (19.9%)	
≥10 units	4 (7.7%)	16 (10.3%)	
End case lactate, mmol/L	5.4 [2.8-8.8]; (0.8-15)	7.1 [5.3-9.9]; (2-16)	.0039†
Δ lactate, mmol/L	3.6 [1.5-5.8]; (−4.8 to 12)	5.1 [3.1-6.9]; (−1.2 to 14)	.0016‡

*NM*, Nautical miles; *n/a*, not available; *pRBCs*, packed red blood cells.

∗ *P* < .001.

† *P* < .05.

‡ *P* < .01.

Postoperatively, hearts stored at 10°C demonstrate similar CI and had similar VIS at the completion of the case (Table 4). At 24 and 72 hours, there was a statistically significant difference in CI, with a slightly greater CI in 10 °C stored hearts (3.2 vs 3.0, *P* = .016; 3.0 vs 2.9, *P* = .037), despite no statistical difference in VIS at similar time points (11.0 vs 12.0, *P* = .21; 6.1 vs 5.3, *P* = .89). Furthermore, hearts stored at 10 °C demonstrate similar pacing support immediately postoperatively (37% vs 35%, *P* = .87), at 24 hours (42% vs 40%, *P* = .75), and at 72 hours (37% vs 24%, *P* = .11).

**Table 4:** Postoperative characteristics and hospital outcomes

Variable	10 °C cooler (n = 52)	Ice (n = 156)	*P* value
Vasoactive-inotropic score			
ICU arrival	18.0 [14-23]; (7-53)	19.0 [14-26]; (5.2-64)	.65
24 h	11.0 [8.5-14]; (3.8-41)	12.0 [9.1-16]; (2.5-36)	.21
72 h	6.1 [4-10]; (0-25)	5.3 [4.0-9.7]; (0-30)	.89
Cardiac index, L/min/m^2^			
ICU arrival	3.0 [2.4-3.5]; (1.5-4.5)	2.6 [2.1-3.3]; (1.1-5.4)	.15
24 h	3.2 [2.8-3.9]; (2.2-5.8)	3.0 [2.5-3.5]; (1-5.6)	.016∗
72 h	3.3 [2.7-3.9]; (2.2-6.3)	2.9 [2.6-3.5]; (1.4-4.7)	.037∗
Pacing required			
ICU arrival	19 (37%)	55 (35%)	.87
24 h	22 (42%)	62 (40%)	.75
72 h	19 (37%)	38 (24%)	.11
Severe PGD	1 (1.9%)	4 (2.6%)	>.9999
ICU LOS, d	6 [5-9]; (3-28)	6 [4-10]; (1-61)	.2853
Hospital LOS, d	15 [13-22]; (8-45)	16 [12-25]; (7-90)	.8749

*ICU*, Intensive care unit; *PGD*, primary graft dysfunction; *LOS*, length of stay.

∗ *P* < .05.

Most notably, hearts stored at 10 °C demonstrate no statistically significant difference in the diagnosis of severe PGD, defined by International Society of Heart and Lung Transplantation guidelines (1.9 vs 2.6%, *P* > .99) (Table 4). There was no difference in ICU (6 vs 6 days, *P* = .28) or hospital LOS (15 vs 16 days, *P* = .87).

### Extended Preservation of Hearts at 10 °C Have Improved Lactate Levels When Compared With Extended Preservation of Hearts Stored on Ice

A further subanalysis of heart allografts with extended preservation time was performed. Hearts with a total ischemic time ≥4 hours were included (n = 23). In total, there was a statistically greater percentage of hearts with extended preservation time stored at 10 °C (44% (23/52) vs 13% (20/156), *P* < .0001) (Table 5). Similarly, there was a larger intraoperative delta lactate levels in the ice storage group (3.7 vs 4.9 mmol/L, *P* = .01). Postoperatively, there was no difference in CI or VIS. ICU LOS (6 vs 7 days, *P* = .94) was similar between temperature groups in all hearts stored for ≥4 hours. An additional subanalysis of allografts stored at >6 hours was performed. There was no ice control group available at that duration. In hearts stored in the 10 °C cooler with ischemic times greater than 6 hours (n = 7), VIS upon ICU arrival, at 24 and 72 hours were 21.7, 11.0, and 10.8, respectively. CI at these same time points was 2.2, 3.0, and 2.8 L/min/m^2^, respectively. The rate of severe PGD was 14%, reflecting a single patient (1/7).

**Table 5:** Prolonged preservation time and advanced donor heart age subanalysis

Ischemic time >240 min	10 °C cooler (n = 23)	Ice (n = 20)	*P* value
Vasoactive-inotropic score			
ICU arrival	21 [14-22]; (7.5-53)	21 [15-28]; (5.3-40)	.42
24 h	11 [9.1-12]; (6.7-24)	12 [9.5-19]; (5.3-36)	.37
72 h	7.1 [5-10]; (0-25)	6.6 [3.3-14]; (1.3-22)	.96
Cardiac index, L/min/m^2^			
ICU arrival	2.6 [2.2-3.3]; (1.6-4.5)	3.3 [2.6-4.0]; (1.1-4.7)	.18
24 h	3.2 [2.8-3.8]; (2.2-4.3)	3.2 [2.7-3.7]; (2.5-4.6)	.73
72 h	3.2 [2.7-3.8]; (2.2-4.7)	3.4 [2.8-3.7]; (2.4-3.9)	.89
End case lactate, mmol/L	5.5 [2.8-9.8]; (1.2-15)	7.6 [5.3-9.7]; (2.9-16)	.20
Δ Lactate, mmol/L	3.7 [1.5-6.1]; (−0.1 to 12)	4.9 [2.8-7.2]; (1.2-13)	.01∗
Severe PGD	1 (4.4%)	2 (10%)	.5900
ICU LOS	6 [6-12]; (3-28)	7 [4-10]; (2-61)	.74

Donor age >40 y old	10 °C cooler (n = 21)	Ice (n = 37)	*P* value
Vasoactive-inotropic score			
ICU arrival	19 [14-26]; (7.5-35)	18 [14-22]; (5.2-39)	.90
24 h	10 [7.6-13]; (3.8-20)	13 [9.5-17]; (3-33)	.067
72 h	7.3 [4.9-12]; (2.5-16)	5 [5-13]; (0-25)	.69
Cardiac index			
ICU arrival	2.9 [2.3-4]; (1.7-4.5)	2.5 [2.0-3.3]; (1.1-4.4)	.23
24 h	3.2 [2.4-3.9]; (2.2-4.3)	2.7 [2.5-3.2]; (1.4-4.2)	.28
72 h	3.6 [2.6-4.3]; (2.2-4.7)	3 [2.6-3.3]; (1.4-3.9)	.21
End case lactate, mmol/L	5.3 [2.3-9.1]; (1.4-15)	6.8 [5.1-10]; (2-16)	.13
Δ Lactate, mmol/L	3.6 [1.1-6.8]; (−0.1 to 11)	5.1 [4.1-6.7]; (0.3-14)	.060
Severe PGD	0	1 (3.2%)	.99
ICU LOS	6 [5-8.3]; (4-28)	5 [4-9]; (1-27)	.58

*ICU*, Intensive care unit; *Δ Lactate*, arterial lactate from the end of surgery-arterial lactate at the time of aortic clamp removal; *PGD*, primary graft dysfunction; *LOS*, length of stay.

∗ *P* < .05.

### Older Donor Hearts Stored at 10 °C Demonstrate Trends But No Significant Difference in Inotrope Requirements and Lactates Compared With Hearts Stored on Ice

An additional subanalysis of donor hearts >40 years old was evaluated (n = 21). There was no difference in the number of hearts >40 years old (40% [21/52] versus 23.7% [37/156], *P* = .69) (Table 5). Similarly, intraoperative delta lactate (3.6 vs 5.1 mmol/L, *P* = .06), postoperative VIS scores immediately postoperatively (19.0 vs 18.0, *P* = .90), at 24 hours (10.0 vs 13.0, *P* = .067) and at 72 hours (7.3 vs 5.0, *P* = .69) and CI postoperatively (2.9 vs 2.5, *P* = .23), at 24 hours (3.2 vs 2.9, *P* = .28), and at 72 hours (3.6 vs 3.0, *P* = .21) were not significantly different in 10 °C hearts, regardless of donor age. ICU length of stay was similar between both groups (6 vs 5 days, *P* = .58).

## Discussion

Cardiac transplantation remains limited by preservation time, creating logistical challenges as the result of timing and geography. Travel distance and preservation time have recently become increasingly prolonged as the result of changes in cardiac allograft allocation in 2018 promoting organ recovery further from the recipient center.^15^ The majority of cardiac allografts continue to be preserved on ice despite commercially available perfusion systems. Given the trend toward further travel distance, the risk of PGD related to ischemic time remains a serious concern.

Transportation temperature has long since been considered a modifiable variable to improve cellular preservation.^16^, ^17^, ^18^ Recently, a commercially available device for hypothermic static storage at 4 to 8 °C for hearts has become available. Industry-sponsored trials comparing cardiac allografts with those preserved with this device are very encouraging and have demonstrated a significant decrease in the rate of severe PGD in both standard and extended criteria donors.^13^^,^^19^ Translational and clinical work in lung transplantation has demonstrated promising at even warmer temperatures. Using 10 °C as the target storage temperature provides benefit with regard to both lung quality and extension of preservation time.^11^^,^^12^ Given the strong scientific signals that 10 °C was the ideal temperature for cellular preservation, we decided to apply this principle to heart allografts and changed our preservation practice to using a 10 °C cooler device for every heart transplant starting in July of 2023.

To analyze whether the benefits of 10 °C storage observed in lungs translated to cardiac allografts, we performed a propensity-matched analysis comparing them with hearts transported on ice. We found that hearts stored at 10 °C were more likely to have longer total ischemic times, with a greater percentage with total ischemic times over 240 minutes. Despite this, the cardiac allografts stored at 10 °C exhibited statistically significant greater cardiac indices with similar VIS, at 24 and 72 hours after surgery. At extended ischemic times, hearts stored at 10 °C had similar postoperative outcomes, with a statistically significant decrease in delta lactate, compared with hearts stored on ice.

We had hypothesized that the rate of severe PGD would be quite low in the 10 °C group. Fortunately, we found that severe PGD was low in both groups, with only 1 case in the 10 °C group (1.9%) versus 5 cases in the ice group (2.6%). In the ice group, this rate is lower than we have historically observed in our institutional data^20^ and is likely an effect of propensity matching.

In extended ischemic time allografts beyond 240 minutes, we observed a rate of severe PGD of 4% in the 10 °C group compared with 10% in the ice group, which was not statistically different. Hospital and ICU length of stay were similar in both groups. VIS and CI were similar between groups. An analysis of cardiac allografts preserved for greater than 360 minutes did demonstrate acceptable CI and VIS scores and a rate of severe PGD of 14%; however, the rate in both groups reflects a single patient between different sized cohorts. An additional subanalysis of donor hearts older than 40 years old also followed similar patterns, albeit with CI trending towards, but not reaching statistical significance.

These data demonstrate that storage of cardiac allografts at 10 °C is at least as good as ice storage, and it may in fact offer benefits as has been reported for lung allografts. Part of this protective effect may be related to maintained cellular repair mechanisms and mitochondrial metabolism and down-regulation of cellular death pathways as shown in animal models.^11^^,^^12^^,^^16^, ^17^, ^18^

Our study population focuses primarily on conventional heart transplantation. Thus, we excluded complex congenital heart patients who are a unique subgroup with a variety of challenges. These patients often have multiple reasons for secondary graft dysfunction: bleeding caused by multiple previous sternotomies, large chest wall and lung collaterals, and abnormal livers causing coagulopathy, abnormal lungs with altered oxygenation/ventilation and pulmonary hypertension, as well as underlying renal dysfunction. These patients often are deconditioned and have little physical reserve. The combination of the aforementioned factors makes them a prohibitive risk for transplantation at most programs, and we believed they should not be compared with conventional recipients in this analysis. Further work to compare postoperative outcomes including severe PGD within these patient populations is of interest.

### Limitations

This is a single-center retrospective analysis and is subject to all the limitations of retrospective studies. A multicenter prospective randomized trial with larger cohorts would better elucidate whether any differences exist. Specifically, we recognize the limitations of a propensity match as a means to approximate a randomized experiment. At our institution, we shifted practice to 10 °C storage exclusively, thus not permitting concurrent comparison between temperature groups. A propensity match, therefore, allows a statistical comparison of a similar patient profile between storage temperature groups. One limitation within propensity match is the identification of matching covariates within a relatively small comparison population. Between February 2020 and July 2023, we identified 197 adult transplant recipients who received an allograft stored on ice who met both inclusion and exclusion criteria, whereas 156 were selected for comparison using our cohort parameters. This proportion of patients matching may result in inexact matching, as opposed to incomplete matching. Subsequently, we acknowledge that despite matching, there is a statistically significant difference in both total ischemic time between groups, whereas hearts stored at 10 °C were more likely to have a longer ischemic time, and with type of procurement, whereas hearts stored on ice had a statistically greater percent of procurement from DBD donors. Longer total ischemic times in the 10 °C storage group may be a result of success with this technique, and our willingness to travel further distances if 10 °C storage was used. There was a statistically significant difference between groups in the percentage of DBD versus DCD hearts. It is unlikely that this impacted our results as we have previously published our institutional experience comparing DBD and DCD allografts and found no difference in outcomes, including severe PGD and 30-day survival, between DCD and DBD hearts.^20^

Furthermore, there are some distinct differences in donor allograft characteristics. There was a greater incidence of HCV + status hearts in our ice cohort. This was likely because of the fact that our institution was one of the earliest users of HCV + cardiac allografts, which gave us access to a high volume of HCV + hearts during the period of 2020 to 2023 when they were not being sought out by other institutions. It is unlikely this influenced these results, as there has been no evidence of a difference in survival outcomes in HCV + heart allografts.^21^ The prevalence of increased cardiopulmonary resuscitation and anoxia/hypoxia in the ice group is likely related to a greater percentage of DBD donors in that group, and as previously mentioned, we have not observed a difference in outcomes between DBD and DCD donors at our institution.^20^

The upper limit of storage time has not yet been reached, as we have successfully transplanted one cardiac allograft after 9 hours using 10 °C storage with no evidence of severe PGD. Future studies should seek to define the limit in preservation duration, as well as examine patient survival and rates of cardiac allograft vasculopathy using this method. Furthermore, mitochondrial and metabolomic profiles of transplanted allografts at 10 °C may elucidate potential mechanisms of cardioprotection.

## Conclusions

This is the first and largest clinical report using 10 °C for preservation of cardiac allografts. Hearts stored in a 10 °C cooler have superior hemodynamic results and lactate profiles compared with hearts stored on ice. In our propensity-matched analysis, we saw no difference in the rate of severe PGD, despite longer ischemic times in the 10 °C cooler group. Further prospective work is needed to confirm our observations and better define the extent of benefit if one exists.

### Webcast

You can watch a Webcast of this AATS meeting presentation by going to: https://www.aats.org/resources/out-of-the-ice-age-preservatio-7036.

**Figure:**
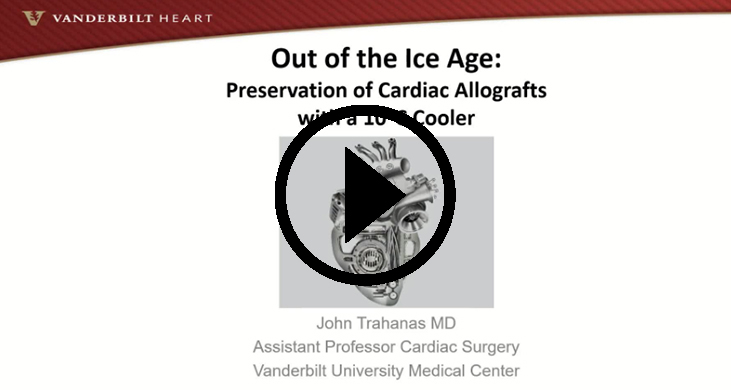

## Conflict of Interest Statement

The authors reported no conflicts of interest.

The *Journal* policy requires editors and reviewers to disclose conflicts of interest and to decline handling or reviewing manuscripts for which they may have a conflict of interest. The editors and reviewers of this article have no conflicts of interest.

## Figures and Tables

**Table E1:** Hearts (n = 7) stored in TRAFEROX cooler for longer than 4 hours reach and maintain 10 °C

Time in cooler	
Median time in cooler, min	335 (256-344)
Cooler temperature	
Median temperature while in cooler, °C (IQR)	8.6 °C (8.4-9.3)
Average temperature when removed from cooler, °C (IQR)	9.85 °C (9.5-10.0)

*IQR*, Interquartile range.

**Table E2:** Comparison of FLIR thermographic at time of arrival to VUMC with TRAFEROX temperature at time of arrival

Sample	FLIR	10 °C cooler	*P* value
1	8.2	7.82	
2	7.275	9.01	
3	7.2	6.13	
4	9.25	8.79	
5	10.8	9.36	
Average	8.545	8.222	.588956785

*VUMC*, Vanderbilt University Medical Center.
